# A growing role for Registry data to guide discussions with patients on their treatment options

**DOI:** 10.1093/jhps/hnae030

**Published:** 2024-12-10

**Authors:** Richard E Field

**Affiliations:** City St George’s, University of London, United Kingdom

In orthopaedic practice, discussions with patients on treatment options often focus on the relative merits of conservative therapies against surgical interventions. Over the course of my career, I have observed management preferences shift from conservative to surgical and visa versa. For example, as an intern, I was expected to set-up and monitor skeletal traction systems for diaphyseal lower limb fractures and the transition to internal fixation was a development that was embraced by patients and surgeons with enthusiasm. I do recall debates whether tibial fractures would be best managed by plating, intramedullary nailing or an external fixator. However, I have no recollection of patients being presented with data on the relative complication rates of the different surgical strategies or the short-, medium-, and long-term clinical outcomes of the different options. In contrast, operations that I observed in my early training to treat children with talipes equinovarus have, for the most part, been superseded by skilled manipulation and conservative treatment [1, 2]. I do recall discussions with patients on the relative merits of conservative verses surgical repair of tendo-Achilles ruptures [3]. These conversations generally ended with the patient asking which option I would choose if I was in their situation. I do not remember an occasion when the patient did not follow medical advice.

Forty years on and we live in an era of shared decision-making [4], Dr Google [5], and patients who sometimes seek multiple opinions before choosing their preferred healthcare provider and treatment pathway. Every surgeon will be familiar with the concerns that patients articulate and the need to provide data that the patient can identify as relevant to their own needs and aspirations [6].

Surgeons advising patients to consider hip replacement are now able to cite implant survival figures from the annual reports of national joint registries that have accumulated data over several decades [7–9], and these surgeons can inform their patients, how long a hip replacement is likely to last depending on the patient’s age and gender as well as the likely influence of the patient’s body mass index and medical comorbidities. Also, the influence of implant-related factors and different surgical approaches to the joint. While hip preservation surgeons can access the annual reports of nonarthroplasty registries [10, 11], such data are derived from the experience of thousands rather than millions of interventions and the reports do not yet enable surgeons to provide their patients with such robust or detailed predictions on the likely benefit of the different hip preserving interventions that could be used to treat their problems.

Nevertheless, national nonarthroplasty hip registries are providing data that can be used to inform patient choice. In this issue, we present a study by Mygind-Klavsen *et al*. [12] using data from the Danish Hip Arthroscopy Registry (DHAR) on patients who have previously undergone periacetabular osteotomy (PAO) for hip dysplasia, and subsequently underwent hip arthroscopy, for residual or persistent symptoms. By way of context, the authors observed that in the following work published by Hartig-Andreasen *et al*. [13], there was a period when combined PAO and arthroscopy was seldom practiced in Denmark. They also noted that Larsen *et al*. [14], had identified from the DHAR dataset that secondary arthroscopy was required for 11% of post-PAO patients.

Mygind-Klavsen’s study provides minimum, 2-year, outcome data of 287 patients for whom hip arthroscopy was used to treat residual intra-articular pathology. This was predominantly residual impingement, labral damage, and articular cartilage damage. While the authors report significant Patient Reported Outcome Measurement (PROM) improvement for this cohort of patients, only 50% achieved a Minimal Clinical Important Difference and only 30% achieved a Patient Acceptable Symptom State. While, this may be an overly harsh assessment of the value of the arthroscopic interventions it is in line with Lukas *et al*.’s systematic review [15] presented in this issue.

Providing patients with guidance on how best to treat symptomatic, borderline, hip dysplasia with concomitant femoroacetabular impingement syndrome remains contentious, and we include a paper from Cabell *et al*. [16] that explores the challenge of helping patients decide whether they would be better treated by PAO with arthroscopy or arthroscopy alone. The authors identify that when surgeons cannot advocate a clear advantage for either of the two treatment options, patient preference become more important and that factors, such as the risk of reoperation and the time to return to sporting activity are the strongest drivers in patient choice.

An additional role for Registries could be to embed research studies. These studies would ‘piggy-back’ on the established data capture pathways. Sohatee *et al*. explore this possibility in their report of a pilot quality improvement study for the UK Non-Arthroplasty Hip Registry (UK-NAHR) [17] to assess the feasibility of this approach. The study focused on how additional support might be deployed to achieve levels of data capture comparable to that seen in conventional randomized controlled trials. While the authors were able to demonstrate a 25% improvement in data capture at 30 days and a 22.4% improvement at 90 days, the overall data capture rates remain below the level that dedicated prospective randomized trials seek to achieve and it remains to be seen whether studies embedded in national registries prove to be cost effective or fail due to data capture rates that compromise the credibility of subsequent data analysis.

We hope that you find the papers in *Journal of Hip Preservation Surgery* (JHPS) Issue 11.3 useful to your practises and that they help you better inform your patients in your discussions on the relative merits of different treatment options.

Finally, we please look at the JHPS website for details of our current calls for submissions for the special issues on The Unhappy/Dissatisfied Hip Preservation Patient: Evaluation and Management and Groin Related Hip Problems.
